# Component Analysis of Four Commercial Brands of Gutta-Percha

**Published:** 2006-07-01

**Authors:** Naghmeh Aminzadeh, Shahram Azimi, Moslem Hagh Shenas

**Affiliations:** 1 *Private Practice, Tehran, Iran*; 2 *Department of Endodontic, Dental School, Azad University of Medical Sciences, Tehran, Iran*; 3 *Polymer Eng., Engineering Research Institute, Tehran, Iran*

**Keywords:** Components, Gutta-Percha Analysis

## Abstract

**INTRODUCTION:** Gutta-Percha, the concrete milky juice of the Sapotaceae family tree, is the most commonly used material for obturation of prepared canal space. Natural 1-4 trans stereochemical structure of Gutta-Percha is taken to a heating- inclusion process of various organic and inorganic elements by manufacturers. The purpose of this study is to detect the presence and percentage of inorganic filler elements and organic phase of various commercial brands and if the locally manufactured brand confonns to standard range of components.

**MATERIALS AND METHODS:** Sample cones, size # 35, from Roeko, Hygienic, DiaDent and AriaDent brands were randomly selected. After burning out in the furnace, organic and inorganic phase percentage and presence of trace elements in each sample were detected and recorded using apparatuses such as SEM, XRF, TGA, IR, NMR, and Ion Chromatography.

**RESULTS:** With slight differences in polymer */ *filler ratio, 78% ± 2% for organic phase, no significant difference was recorded among these brands. SEM analysis detected Zinc, Barium, and Sulfur in Hygienic, DiaDent, and AriaDent in descending order; in the last two Silicones was also traced, while Zinc was the only element to be identified in Roeko.

**CONCLUSION:** No significant chemical and structural differences among four commercial brands were detected.

## INTRODUCTION

Gutta-Percha, is one of the first natural organic polymers to be exploited by man, sharing isoprene monomer, 104-106 molecular weight (1), as the basic structure (2), Gutta-Percha is the trans isomer of polyisoprene differing dramatically in its tensile properties from natural rubber .The natural rubber (cis-isomer) is essentially amorphous and Gutta-Percha is approximately 60 % crystalline. This fact largely accounts for the difference in their respective mechanical properties (2). Gutta­ Percha polymeric molecule has two distinct interconvertible alpha and beta crystalline forms, but not convertible into natural rubber. Both types differ only in single bond configuration and molecular repeat distance. Slow cooling of the heated Gutta-Percha gives rise to alpha chains, while rapid cooling ends in beta chai ns. There is apparently no difference in the mechanical properties of A and B types but there are thermal and volumetric differences (3,4). Pure Gutta-Percha is rigid at ordinary temperature, becomes pliable at 25°-30°C, softens at 60°C and melts at 100°C with partial decomposition (5). Its plasticity at a relatively low temperature made it useful in dentistry (1).

Friedman et al. compared the composition and mechanical properties of five commercial Premier, Mynol, Indian Head, Dent-O-Lux, and Tempryte brands (2). Marciano also worked on the chemical composition of ten commercial brands such as Hygienic, Mynol, Roeko, Detrey, Becht, Septodent, Medio-Dentaire, SPAD, IFKER, and Endoset (1) (Table 1).

**Table 1:** The maximum and minimum amounts of component percentage of commercial gutta­percha in previous studies

	**Gutta-Percha**	**Zinc Oxide**	**Heavy Metal Sulfates**	**Waxes and/ or Resins**
**Friedman 1975**	18/9-21/8	59/1-75/3	1/5-17/3	1/0-4/1
**Friedman 1977**	18/9-21/8	59/1-75/3	1/5-17/3	1/0-4/1
**Marciano 1989**	17/73-45/85	36/55-74/57	2/28-31123	-

The major content of cone is Zinc Oxide, having some chemical interaction with Gutta­ Percha, which seems to be responsible for some antibacterial properties and functions as a vulcanizing agent (2,6). High levels of Zinc Oxide tend to increase brittleness and to reduce flow of the material (5). Plasticity of the cone is due to relatively low content of Gutta-Percha where as increase in that proportion leads to brittleness of the cone (1). Tensile and yield strength are significantly correlated to gutta­ percha percentage; however other characteristics such as resilience, elastic modulus, and ultimate tensile strength are not related (5). The addition of small amounts of plasticizers increases the flexibility and compactness of the cone (7).

## MATERIALS AND METHODS

One box of randomly purchased size 35 Gutta­ Percha from four brands such as AriaDent, Roeko, Hygienic, and DiaDent were used. In order to obtain the inorganic proportion of each single cone of Gutta-Percha, cones were separately burnet in furnace at temperature of 600-700°C for 3 hours and then net weight of ash after evaporation of organic contents was calculated as the following: IEP= (Ash+BW)-(BNW)/ (Bowl +GPCW)-(BNW)

*(TEP: Inorganic Element Percentage, BW: Bowl weight, BNW: Bowl Net Weight, GPCW: Gutta-Percha Cone Weight)*

**Figure 1:**
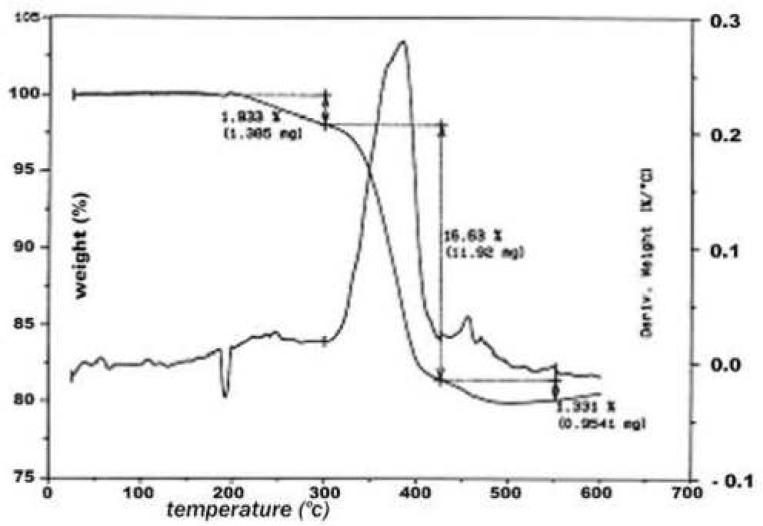
Organic and inorganic ingredients of Hygienic gutta-percha cones by TGA analysis.

The results were compared to the findings of the ThermoGravimetry Analysis (TGA) device (951 Du Pont, USA) in which one milligram of each sample was heated up to 520°C with N_2_0 gas and then up to 700°C with oxygen, and the net weight of the residual ash was the inorganic content in each sample (Figure 1).

To identify the non-metallic and metallic elements and their semi-quantitative proportion in inorganic fillers, samples were exposed to electrons in vacuum chamber of SEM (Cambridge, UK) and detected by DEX and WEX receptors.

To find out the semi-quantitative percentage of metallic oxides we used X-Ray Fluorescence or XRF device (EX90A, Jeol, Japan), and also Ion Chromatography (HIC6A, Shimadzu, Japan) helped to divide the inorganic part as anionic and cationic structures.

Finally, infrared wave lengths or IR device (IFS88, Bruker, Germany) on monomer chains and magnetic field in Nuclear Magnetic Resonance or NMR (9050Q, Philips) were used to identify the organic phase within each sample.

## RESULTS

After burning the samples in Furnace, the average percentage of total filler ingredients were 78 % ± 2 % for all samples.

**Figure 2:**
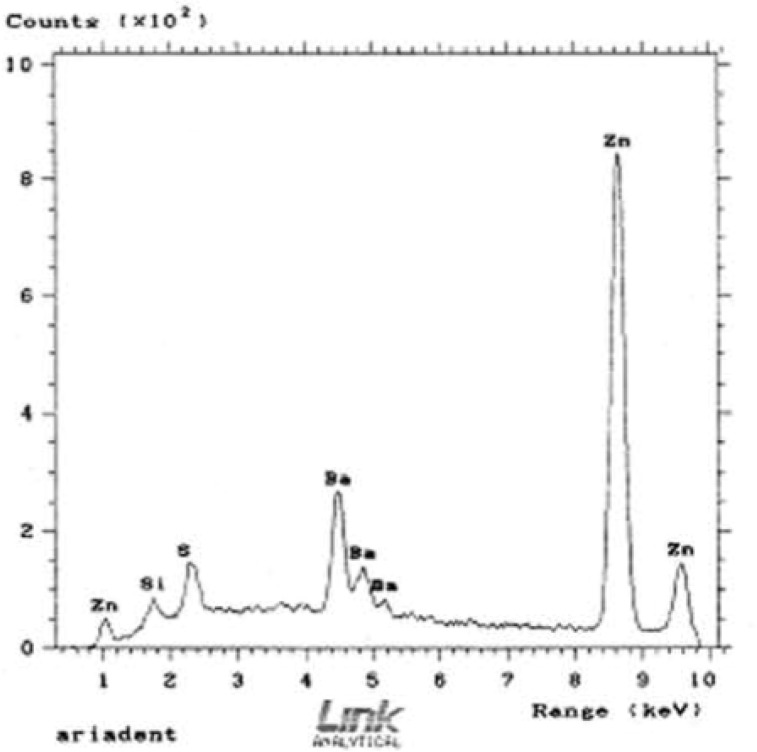
SEM chemical analysis of AriaDent gutta-percha cones.

SEM chemical anal ysis detected Zinc, Barium and Sulfur in Hygienic, DiaDent and AriaDent in descending order (Figure 2), with Silicone traced in the last two brands, while Zinc was the only element to be identified in Roeko.

The results of Infrared exposure to monomer chains were identical, and demonstrated the bonding groups such as CH_2_ (peak at 1437), CH_3_ (peak at 1366), C = C (peak at 1634) and C-H (peak at 2855) which were the same in all samples.

The findings in NMR analysis, for protons of methylene (-CH_3_), ethylene (-CH_2_), and broad singlets of (C-H) were identical in all samples as shown below:

1/65 ppm (3H, singlet, CH_3_)

2/05 ppm (4H, singlet, CH_2_)

*51*I 0 ppm (1H, broad singlet, CH)

**Figure 3:**
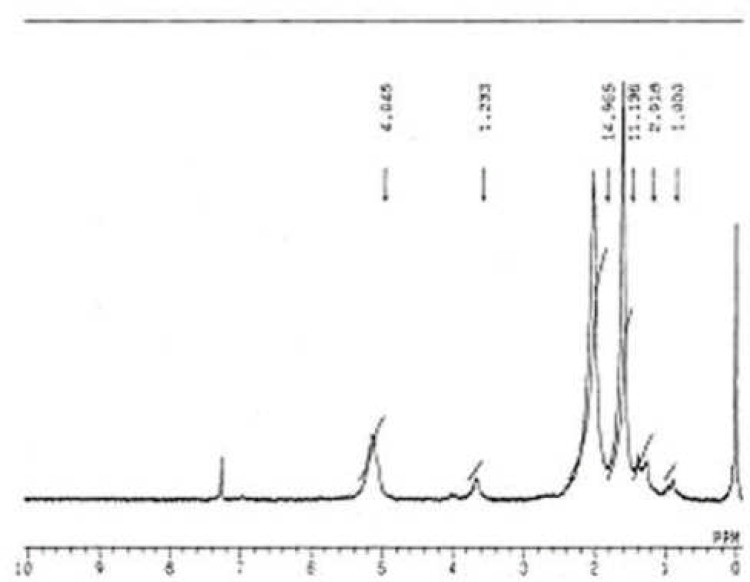
NMR spectrum of gutta-percha in Hygienic points.

The NMR results indicated slightly increased hydrocarbonic components such as waxes in Hygienic cones (Figure 3).

The findings of XRF analysis is shown in Table 2. These results revealed the semi­ quantitative percentage of metallic oxides in gutta-percha cones.

Although Ion Chromatography analysis is a very precise test, but due to low dissolution of Barium Sulfate and Zinc Oxide in water and acid, only few inorganic compounds such as Chlorine (-Cl), Nitrate (-N0_3_) and Sulfate (­SO_4_) were detected because of their absolute dissolution in water.

## DISCUSSION

Polyisoprene chain may exist four stereochemical 1-2, 3-4 and 1-4 cis and 1-4 trans types. Not recording organic phase peaks by NMR device at 5.82, 5.00, and 4.80 ppm indicated the absence of 1-2, 3-4 structures in Gutta-Percha, and concluded that natural Gutta-Percha has no grafting double bonds (8). Detecting minor concentrations of 1-4 cis (1.80 ppm) indicated that the majority of commercial points contained 1-4 trans (5.37 ppm) structure which was identical to that of natural Gutta­ Percha and the structure was not altered by the heating process during its preparation (8). Recording a mean peak of 1.30 ppm might indicated the presence of wax (hydrocarbon) in the structure of all groups, although peaks at 1.00, 1.30 and 3.70 ppm in Hygienic cones Showed slightly higher percentage of waxes than the other groups (Figure 3).

Interestingly, Marciano had found great differences in the chemical structure of not only various commercial brands but also among various sizes of one individual brand which probably explained the diversity in enthalpic behavior of Gutta-Percha points when heated (1).

Since only size # 35 of each brand was collected in our study, we were not able to investigate such differences among sizes of a single brand.

Our findings for the average Gutta-Percha content in four commercial brands are in conformity with those of Marciano and Friedman (1,2,5,8).

**Table 2:** XRF analysis of the amounts of inorganic ingredients in four brands of commercial gutta-percha points

**An** **a** **l** **y** **te**	**AriaDent**	**DiaDent**	**H** **y** **gienic**	**R** **o** **e** **ko**
**Na**_2_ **O**	%11.5	%12.2	%10.9	%14.2
**MgO**	%4.4	%5.2	%2.1	%1.7
**AL**_2_ **O**_3_	%2.3	%2.5	%7.1	%2.2
**SiO**_2_	%4.1	%3.8	%2.2	%2
**SO**_3_	%4.5	%3.3	%3.6	%1.3
**K**_2_ **O**	710ppm	880ppm	990ppm	770ppm
**CaO**	%0.5	%0.4	%0.5	%0.3
**TiO**_2_	%2.3	%4.3	%2.4	%0.6
**Fe**_2_ **O**_3_	%0.2	%0.2	%0.2	%0.4
**NiO**	420ppm	330ppm	400ppm	270ppm
**BaO**	%7.4	%7.7	%6.6	%2.1
**PbO**	%0.2	%0.2	%0.2	%0.1
**SnO**	470ppm	480ppm	520ppm	430ppm
**Cl**	710ppm	770ppm	-	650ppm
**ZnO**	%61.1	%58.4	%63	%73.5
**MnO**	-	240ppm	230ppm	-
**SrO**	-	980ppm	900ppm	-
**Ba**	%6.628	%6.896	%5.91	%1.88
**SO**_4_	%4.63	%4.82	%4.22	%1.31
**BaSO**_4_	%11.26	%11.72	%10.13	%3.20
**Na**	%8.53	%9.05	%8.09	%10.53

There seems to be an inverse relationship between Zinc Oxide*/*metallic sulfates and Gutta-Percha*/*wax concentrations. Barium Sulfate was the least while Zinc Oxide was recorded as the highest in Roeko, and vice versa in DiaDent group which was also in accord with findings of Friedman (6).

Addition of trace amounts of Silicone seemed to increase the hardness, thermal, chemical and frictional resistance and enhanced its structural stability. Addition of Titanium Oxide increased the thermal stability and enhanced the dye penetration into the matrix which was Erythrosin for Gutta-Percha cones (8); therefore imparting the brighter color to the cone is achieved.

## CONCLUSION

Recording range of 78% ± 2% for organic phase of all four commercial brands and non-significant differences for their contents of trace elements, no specific structural differences were found among locally manufactured AriaDent and other brands.
